# Maternal high-protein diet modulates hepatic growth axis in weaning piglets by reprogramming the *IGFBP*-*3* gene

**DOI:** 10.1007/s00394-019-02097-z

**Published:** 2019-09-30

**Authors:** Rihua Cong, Xiaoli Qu, Hui Zhang, Yongling Hu, Silin Ye, Demin Cai, Xian Li, Hao-Yu Liu

**Affiliations:** 1 College of Veterinary Medicine, Northwest A and F University, Yangling, 712100 Shannxi China; 2 School of Life Sciences, Zhengzhou University, Zhengzhou, 450001 China; 3 Department of Biochemistry and Molecular Medicine, University of California at Davis, Sacramento, 95817 CA USA; 4 Department of Medical Cell Biology, Uppsala University, SE-75123 Uppsala, Sweden; 5 College of Animal Science and Technology, Yangzhou University, Yangzhou, 225009 Jiangsu China

**Keywords:** High-protein diet, Meishan piglets, DNA methylation, Histone modification, Growth axis, IGFBP-3

## Abstract

**Purpose:**

The aim of this study was to investigate the effects of maternal high dietary protein intake on the hepatic growth axis in offspring.

**Methods:**

Fourteen primiparous purebred Meishan sows were fed either a standard-protein (SP, *n* = 7) diet or a high-protei*n* (HP, 150% of SP, *n* = 7) diet during pregnancy. Offspring (one male and one female per group, *n* = 14) on day 70 of the embryonic stage and on days 1, 35 and 180 after birth were selected, weighed and killed. Serum samples were analyzed for Tch, insulin and insulin-like growth factor-binding protein 3 (IGFBP-3) levels. Liver samples were analyzed for *IGFBP*-*3* and *IGF*-*I* mRNA expression by qRT-PCR and for IGFBP-3, IGF1R and growth hormone receptor (GHR) protein expression by Western blotting. The underlying mechanism of IGFBP-3 regulation was determined by methylated DNA immunoprecipitation (MeDIP) and chromatin immunoprecipitation (ChIP).

**Results:**

High-protein exposure resulted in significantly higher body and liver weights of piglets, and it increased their serum T3 and T4 levels at birth and/or at weaning. Furthermore, the IGFBP-3 protein content in the liver and serum was significantly reduced in the HP-exposed weaning piglets, whereas at the transcriptional level *IGFBP*-*3* mRNA expression was downregulated in the livers of HP group piglets. Finally, DNA hypermethylation and higher enrichment of the histone repressive marks H3K27me3 and H3K9me3 were observed.

**Conclusions:**

Taken together, these results suggest that a maternal high-protein diet during gestation epigenetically reprograms *IGFBP*-*3* gene expression to modulate the hepatic growth axis in weaning piglets.

## Introduction

The insulin-like growth factor (IGF) system consists of two IGF ligands (IGF- I and IGF-II), two IGF receptors (IGF-1R and IGF-2R), and a group of at least six IGF-binding proteins (IGFBP-1 to 6) [1]. These components are found in a variety of tissues or bodily fluids [2] and act through endocrine, paracrine, and/or autocrine modes of action [3]. As a result, the IGF system, with all components working together, regulates biological processes, including cell proliferation, differentiation, survival and apoptosis that are essential to life [4, 5]. In particular, the IGF system plays an important role in the link between nutrition and postnatal growth and development [6]. For example, fetal nutrient supply determines the fetal IGF axis [7–9] and can continue shaping the IGF axis after birth, as revealed in rodents [10] and humans [11]. It has been shown that a maternal low-protein diet (LPD) is associated with a reduced fetal liver weight in rats [12, 13]. The livers of progeny whose mothers were fed a high-protein diet during gestation were 8.3% heavier [12, 13]. Accordingly, a maternal LPD causes a decrease in fetal hepatic *IGF*-*I* gene expression, but increases IGFBP levels in fetal plasma and liver, which may contribute to fetal growth retardation, as observed in rats [14–18]. Previous studies have demonstrated that gene expression is associated with progesterone, IGF-I and IGFBP-5, which are upregulated in the fetus in response to maternal high-protein intake [17, 19, 20]. In the case of large litters in pigs, the excess of dietary protein seems to be beneficial for preventing intrauterine growth retardation (IUGR) resulting from intrauterine crowding [20]. Notably, recent studies have revealed that maternal protein supplementation during gestation affects human fetal body weight independent of IGF-I [21]. In addition, hormones, especially those from the thyroid, are thought to be key players connecting the IGF growth axis and maternal factors/fetal nutrient supply. Thyroid hormones can regulate IGF-I production by activating the hepatic growth hormone receptor (GHR) [22, 23], and they are sensitive to maternal nutritional reprogramming [24, 25]. Nevertheless, the exact nature of these events is still unclear and warrants further exploration.

Interestingly, it has been well documented that epigenetic regulation modifies fetal gene expression during maternal nutrition programming. For instance, in cancer research, the IGFBP-3 gene is described to be vulnerable to epigenetic regulation, including DNA methylation and histone modification [26]. It is worth mentioning that IGFBP-3 can block IGF-I and IGF-II from binding to their receptors and therefore plays an important role in cell proliferation stimulation. However, whether maternal dietary intervention affects gene expression in offspring via epigenetics has not been well explored. Nawathe et al. showed that DNA methylation is involved in changes of gene expression in the IGF axis in fetal growth disorders [27], while maternal undernutrition alters hepatic metabolic gene transcription via DNA methylation and the modification of histones, including H3K4me3, H3K27me3 and H3K9me1 [28, 29]. Moreover, modifications of H3K14ac and H3K9me3 are found to occur in fetal liver lipid metabolism when exposed to a high-fat diet in utero [30].

Intriguingly, genes involved in the liver cell cycle and growth in newborn piglets are downregulated by histone modification when the dams are exposed to dietary supplementation with methyl donors [31]. Building upon these studies, we hypothesized that epigenetic events during maternal nutrition programming may play a crucial role in modifying the liver IGF-I/IGFBP-3 growth axis in offspring.

Meishan (MS), a Chinese indigenous pig breed, pigs were used in the present study as an animal model to delineate the impact of a maternal high-protein diet on the hepatic IGF axis of offspring piglets at different life stages. Blood and liver samples were taken from sows at 70 days gestation, from piglets at birth, from weaned piglets at 35 days, and from fattened pigs at 180 days. We aimed to explore the possible epigenetic mechanisms on the regulation of specific genes involved in the IGF-I/IGFBP-3 pathway in the liver.

## Methods

### Animals and sampling

The experimental protocol was approved by the Animal Ethics Committee of Northwest A & F University. All procedures complied with the ‘Guidelines for the Ethical Treatment of Experimental Animals’ (2006) No. 398 set by the Ministry of Science and Technology, China. Eighteen primiparous purebred Meishan gilts were obtained from the National Meishan Pig Preservation and Breeding Farm at Jiangsu Polytechnic College of Agriculture and Forestry, Jurong, Jiangsu Province, P. R. China. They were assigned randomly to a high-protein (HP) or standard-protein (SP) group.

**Fig. 1:**
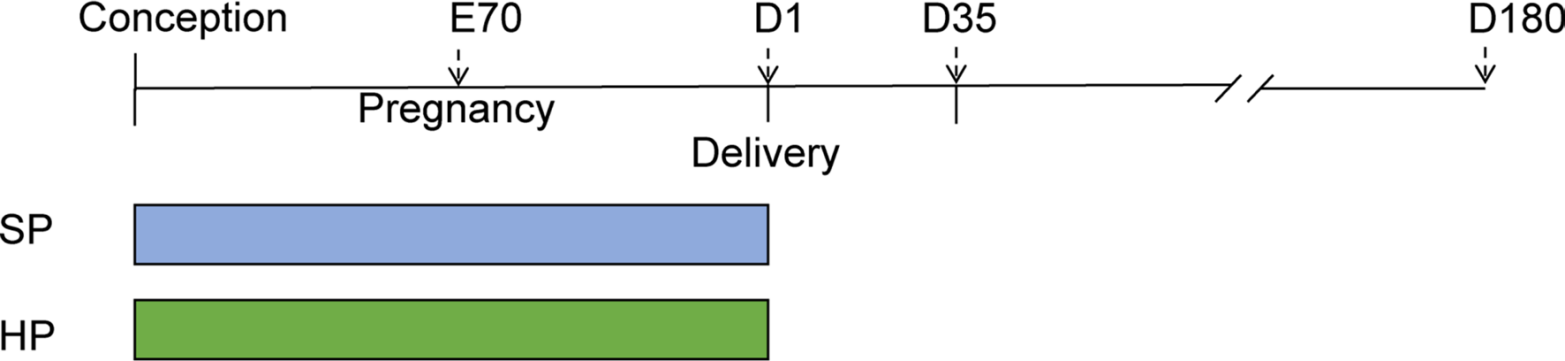
Experimental design. Standard-protein (SP) or high-protein (HP) diets were fed to dams during pregnancy. Offspring of different dietary groups were analyzed at embryonic day 70 (E70) and postnatal day 1 (D1), day 35 (D35) and day 180 (D180). *SP* standard protein during pregnancy and lactation, *HP* high-protein diet during pregnancy and lactation

Sows were fed diets containing either 14% crude protein in the HP group or 7% crude protein in the SP group (Table 1). The dietary treatment started from the first observation of estrus and artificial insemination was performed at the second estrus. A mixture of semen samples obtained from two littermate boars was used for artificial insemination, and the fertilization rate was not influenced by maternal dietary treatment. Sows were fed twice daily (0800 and 1400 h) with rations of 1.8 kg/day during gestation. As shown in Fig. 1, we randomly selected piglets on day 70 of the embryonic stage (E70) and on the 1st (D1), 35th (D35) and 180th (D180) days. At each stage, one male (*n* = 7) and one female (*n* = 7) piglet (per experimental group) with a mean body weight ± 10% were selected from each litter and killed. Blood was collected, followed by immediate serum preparation, and the liver (without the gall bladder) samples were snap frozen in liquid nitrogen immediately and stored at − 80 °C for further analysis.

**Table 1:** Composition and nutrient content of the experimental diet

Ingredient %	HP	SP
Corn	61	55.8
Soybean meal	17	
Bran	12	15
Bone meal	1	0.5
Corn sugar	5	22
CaSPO_4_		0.7
Fiber^a^		1
Attapulgite		1
Premix^b^	4	4
*Calculated composition*		
Digestible energy, MJ/kg	13.1	13.1
Crude protein %	14	6.9
Crude fiber %	2.8	2.6
Ca %	1.2	1.2
*P* %	0.4	0.4

^a^The fiber concentrate ARBOCEL was purchased from JRS (Germany)

^b^The premix contains (per kilogram): vitamin A: 240,000 IU; vitamin D3: 60,000 IU; vitamin E: 720 IU; vitamin K3: 30 mg; vitamin B1: 30 mg; vitamin B2: 120 mg; vitamin B6: 60 mg; vitamin B12: 360 mg; niacin: 600 mg; pantothenic acid: 300 mg; folic acid: 6 mg; manganese sulfate: 1.0 g; zinc oxide: 2.5 g; iron sulfate: 4.0 g; copper sulfate: 4.0 g; sodium selenite: 6 mg; calcium: 150 g; phosphorus: 15 g; sodium chloride: 40 g

### Tch, insulin and IGFBP-3 in serum

Serum concentrations of insulin (F01PZB), T3 (A01PZB) and T4 (A02PZB) were measured using specific commercial RIA kits (Beijing North Institute of Biological Technology) with assay sensitivities of 5 μIU/L, 0.25 mg/L and 5 mg/L, respectively. The intra- and inter-assay variations were either 10% or 15% for the kit used. Serum IGFBP-3 levels were measured using a commercial ELISA kit (Abcam, ab100541) following the manufacturer’s instructions.

### Total RNA isolation and mRNA quantification

Total RNA was isolated from liver samples using TRIzol reagent (Tiangen Biotech Co., Ltd., Beijing, China). The iScript cDNA Synthesis Kit (Promega, Madison, WI, USA) was used to synthesize cDNA from 2 μg of total RNA from each sample according to the manufacturer’s instructions. Two microliters of diluted cDNA (1:50) was used for quantitative real-time PCR. The primer sequences are shown in Table 2 and were synthesized by Invitrogen (Shanghai, China). Real-time PCR was performed with Mx3000P (Stratagene, USA). The technical variations were normalized to GAPDH as an internal control. The specificity of amplification was determined by melting curve analysis and PCR product sequencing.

**Table 2:** Nucleotide sequences of specific primers

Gene name	Accession no.	Primer sequence (5′ → 3′)	Size (bp)
*GAPDH*	AF017079	F^*^: GGACTCATGACCACAGTCCAT R^*^: TCAGGTCCACAACCG ACACGT	220
*IGF*-*I*	DQ784687	F: ATTTCTTGAAGGTAAAGATGCA R: CAGCCCCACAGAGGGTCTCA	117
*IGFBP*-*3*	AF085482	F: GACACGCTGAACCACCTCA R: CGTACTTATCCACGCACCAG	151
IGFBP-3 Promoter	NM_001005156.1	F: AAATGGAGGACACGCTGAAC R: TACTTATCCACGCACCAGCA	157

^*^F  forward, *R*  reverse

### Tissue protein extraction and Western blot analysis

Total cellular protein and nuclear protein were extracted from 100 mg frozen liver tissue as described previously [32, 33]. The protein concentration was measured with a Pierce BCA Protein Assay Kit (Thermo Scientific, USA). Western blot analysis for IGF-1R (ab226871, Abcam, UK, diluted 1:100), IGFBP-3 (ab231034, Abcam, UK, diluted 1:100) and GHR (ab202964, Abcam, UK, diluted 1:200) was carried out according to the recommended protocols provided by the manufacturer.

### Methylated DNA immunoprecipitation (MeDIP)

Methylated DNA immunoprecipitation (MeDIP) analysis was performed as previously described with some modifications [12]. Briefly, genomic DNA of the liver was sonicated and heat denatured to generate single- stranded DNA. A mouse monoclonal antibody against 5-methyl cytosine (ab10805, Abcam) was used for immune-precipitating methylated DNA segments. Pretreated G protein agarose beads (40 μL, 50% slurry, sc-2003, Santa Cruz Biotechnology) were used to capture the precipitated immune complexes, and purified MeDIP DNA was used to amplify the proximal promoter sequences of the target genes by RT-PCR. The CpG islands on the promoter of the *IGFBP*-*3* gene was assessed by Methyl Primer Express v1.0 (Applied Biosystems, USA) using the following criteria:  %GC > 50%, length > 200 bp, and CpG observed/CpG expected > 0.6. A pair of negative control primers was used to amplify a promoter region without CpG sites as the internal control, and the MeDIP results were calculated relative to the internal control. The specific primers are shown in Table 2.

### Chromatin immunoprecipitation (ChIP)

ChIP analysis was performed according to previous publications with some modifications [34]. Briefly, 200 mg frozen liver samples were ground in liquid nitrogen, resuspended in PBS containing a protease inhibitor cocktail (Roche Diagnostics GmbH, Mannheim, Germany) and cross-linked in 1% formaldehyde for 10 min at room temperature. The cross-linking reaction was stopped by 2.5 M glycine while rotating for 10 min at room temperature. The pellets were washed using PBS and rinsed with sodium dodecyl sulfate (SDS) lysis buffer (50 M Tris–HCl pH 8.1, 10 mM EDTA, 1% SDS) containing protease inhibitors. The cross-linked samples were sonicated for 10 min on ice with 10 s on/off intervals (Sonics Vibra, USA). The samples were then centrifuged at 12,000 rpm for 10 min at 4 °C to remove cell debris from the crude chromatin preparations. The average length of the sonicated chromatin was approximately 500 bp, as determined by 1% agarose gel. The protein–DNA complex was diluted in ChIP dilution buffer, precleared with salmon sperm DNA/G protein agarose beads (60 μl, 50% slurry, Biyuntian, Biotechnology, China) and incubated with 2 μg of the respective antibody [histone H3 antibody, ab1791, Abcam; anti-acetyl-histone H3, 06-599, Millipore; trimethyl -histone H3K9, ab8898, Abcam; trimethyl-histone H3K27 (Lys27), 17-622, Millipore; trimethyl-histone H3K4 antibody, ab1012, Abcam] overnight at 4 °C. A negative control was included without adding an antibody. G protein agarose beads (120 μl, 50% slurry) were added to capture the immunoprecipitated chromatin complexes. The pellets containing immunoprecipitated complexes were sequentially washed, and the antibody/protein/DNA complexes were eluted from protein G agarose beads. Finally, reverse cross-linking was performed at 65 °C for 5 h to release DNA fragments from the immunoprecipitated complex, and the ChIPed-DNA was purified.

### Statistical analysis

All data are presented as the mean ± S.E.M. and were analyzed using an independent-samples *t* test with SPSS 22.0. The 2 − ΔΔCt method was used to analyze the RT-PCR data expressed as the fold change relative to the SP group. Since none of the detected parameters showed sex disparities, males and females were grouped together, resulting in *n* = 14 in each group. The numbers of piglets for the different measurements achieved statistical power of 0.95 (using the *t* test) between the groups, with the relevant standard errors. Differences were considered significant at *p *< 0.05.

## Results

### A maternal high-protein diet increases the body and liver weight of piglets and alters serum parameters

**Fig. 2:**
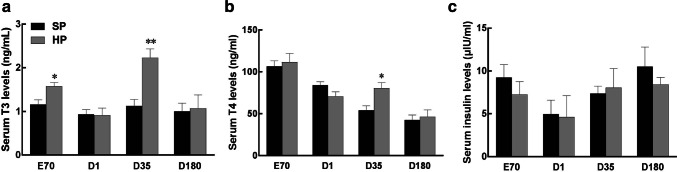
Serum concentrations of T3 (**a**), T4 (**b**) and insulin (**c**) in piglets at embryonic day 70 (E70) and postnatal day 1 (D1), day 35 (D35) and day 180 (D180). Values are mean ± S.E.M., *n* = 14, *p < 0.05, **p < 0.01

As shown in Table 3, piglets derived from HP diet-fed sows exhibited significantly higher body weight (*p *< 0.01) and liver weight (*p *< 0.01) at birth and at weaning than the piglets in the SP group. The adult body weight was similar between treatments. Additionally, a significantly increased serum concentration of T3 and T4 was observed at weaning (Fig. 2a, b, *p *< 0.05). However, the maternal HP diet did not affect serum insulin levels in offspring at any stage (Fig. 2c).

**Table 3:** Body weight, liver weight and liver index of the offspring piglets at embryonic 70 (E70), postnatal 1 (D1), 35 (D35) and 180 (D180) days

Parameter	D1		D35		D180	
	SP	HP	SP	HP	SP	HP
BW kg	0.84 ± 0.02	0.97 ± 0.03^**^	5.02 ± 0.35	6.62 ± 0.46^*^	61.42 ± 2.39	66.98 ± 2.03
LW g	21.6 ± 0.66	26.8 ± 1.84^**^	130.8 ± 5.70	172.5 ± 7.25^**^	966.1 ± 75.72	951.2 ± 62.23
Liver index g/kg	27.4 ± 1.57	25.1 ± 0.98	26.6 ± 1.52	26.7 ± 1.30	14.0 ± 1.02	15.9 ± 1.40

Values are mean ± S.E.M., n = 14. ^*^p < 0.05. ^**^p < 0.01 compared to SP

### A maternal high-protein diet reduces liver IGFBP-3 protein content and serum IGFBP-3 level in weaning piglets

**Fig. 3:**
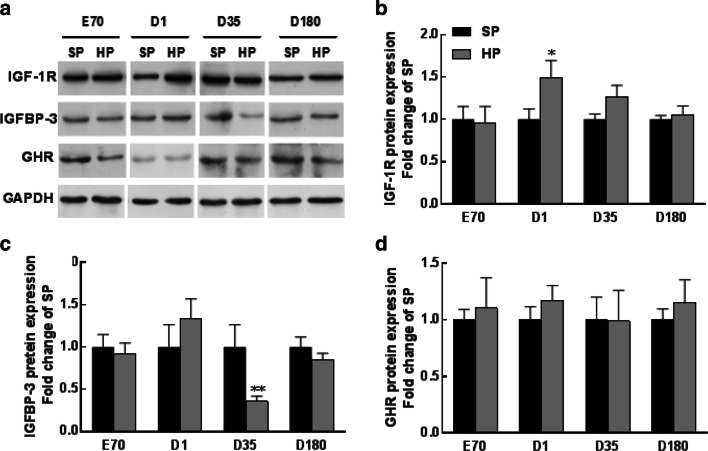
Western blotting analysis (**a**) and graphic summary of IGF-1R (**b**), IGFBP-3 (**c**) and GHR (**d**) protein contents in piglets at embryonic day 70 (E70) and postnatal day 1 (D1), day 35 (D35) and day 180 (D180). Values are mean ± S.E.M., *n* = 14, *p < 0.05, **p < 0.01

**Fig. 4:**
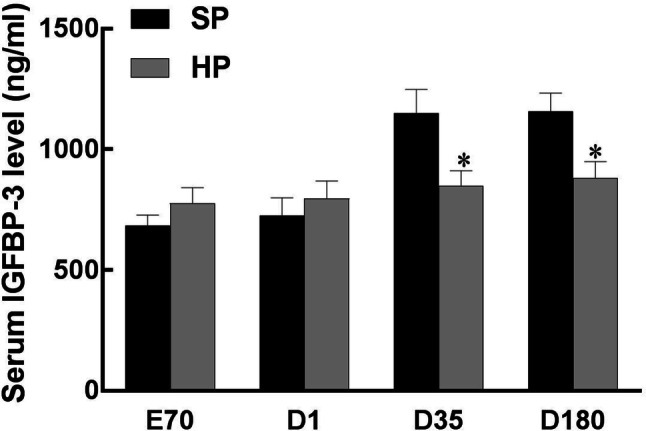
ELISA analysis of IGFBP-3 levels in the serum of piglets at embryonic day 70 (E70) and postnatal day 1 (D1), day 35 (D35) and day 180 (D180). Values are mean ± S.E.M., *n* = 14, *p < 0.05

To identify the possible events that caused the growth effects in piglets, we performed Western blotting to detect the key proteins involved in the liver growth axis. IGF-1R protein content was significantly elevated at birth, whereas IGFBP-3 protein content was markedly reduced at weaning in the HP-exposed piglets (Fig. 3a–c). The GHR protein content was unaltered at any of these time points in the offspring’s life (Fig. 3a and d). Furthermore, consistent with the decreased IGFBP-3 protein content, we revealed that the serum IGFBP-3 level was also decreased in HP-exposed weaning piglets, as well as in adult offspring (Fig. 4).

### Maternal high-protein diet downregulates liver IGFBP-3 transcript

**Fig. 5:**
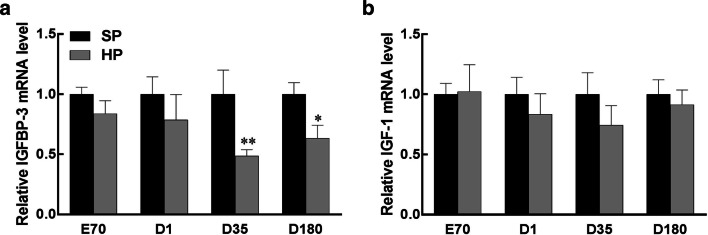
Relative IGFBP-3 (**a**) and IGF-I (**b**) mRNA expression measured by qRT-PCR in the liver of piglets at embryonic day 70 (E70) and postnatal day 1 (D1), day 35 (D35) and day 180 (D180). Values are mean ± S.E.M., *n* = 14. * *p* < 0.05, ** *p* < 0.01

Given the significant changes in hepatic protein contents of IGF-1R and IGFBP-3 in HP-exposed piglets, qRT-PCR was conducted to validate the transcriptional level of these two genes. *IGFBP*-*3* mRNA was significantly downregulated in the livers of weaning and adult piglets as a result of HP treatment (Fig. 5a). In contrast, *IGF*-*I* gene expression at the transcriptional level in piglets was not affected by treatments at any time point (Fig. 5b).

### Epigenetic regulation of the IGFBP-3 gene in HP-exposed weaning piglets

**Fig. 6:**
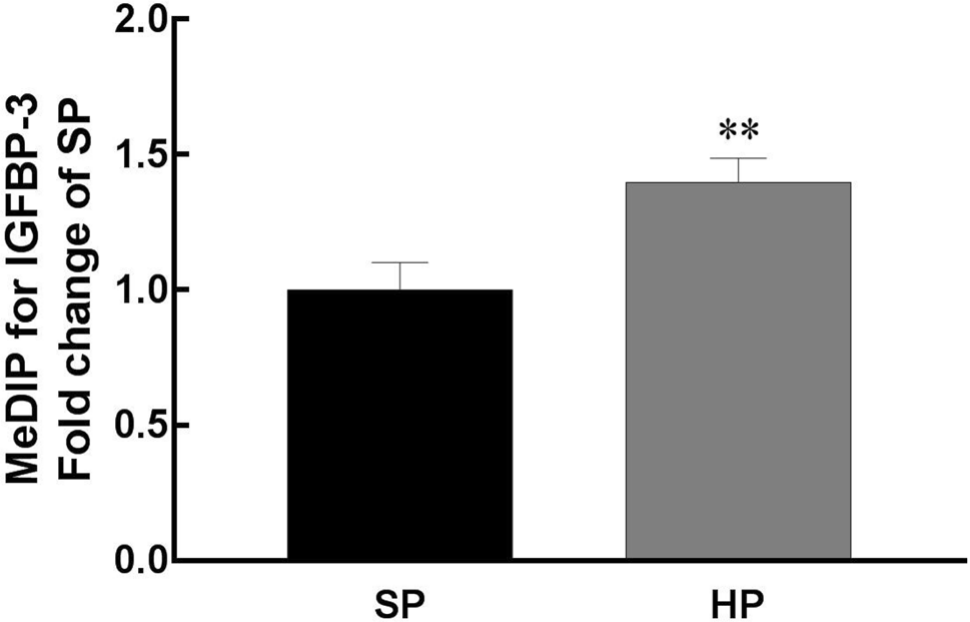
Methylated DNA immunoprecipitation (MeDIP) analysis of DNA methylation on hepatic *IGFBP*-*3* gene promoter in weaning piglets. Values are mean ± S.E.M., *n* = 14. ^**^p < 0.01

**Fig. 7:**
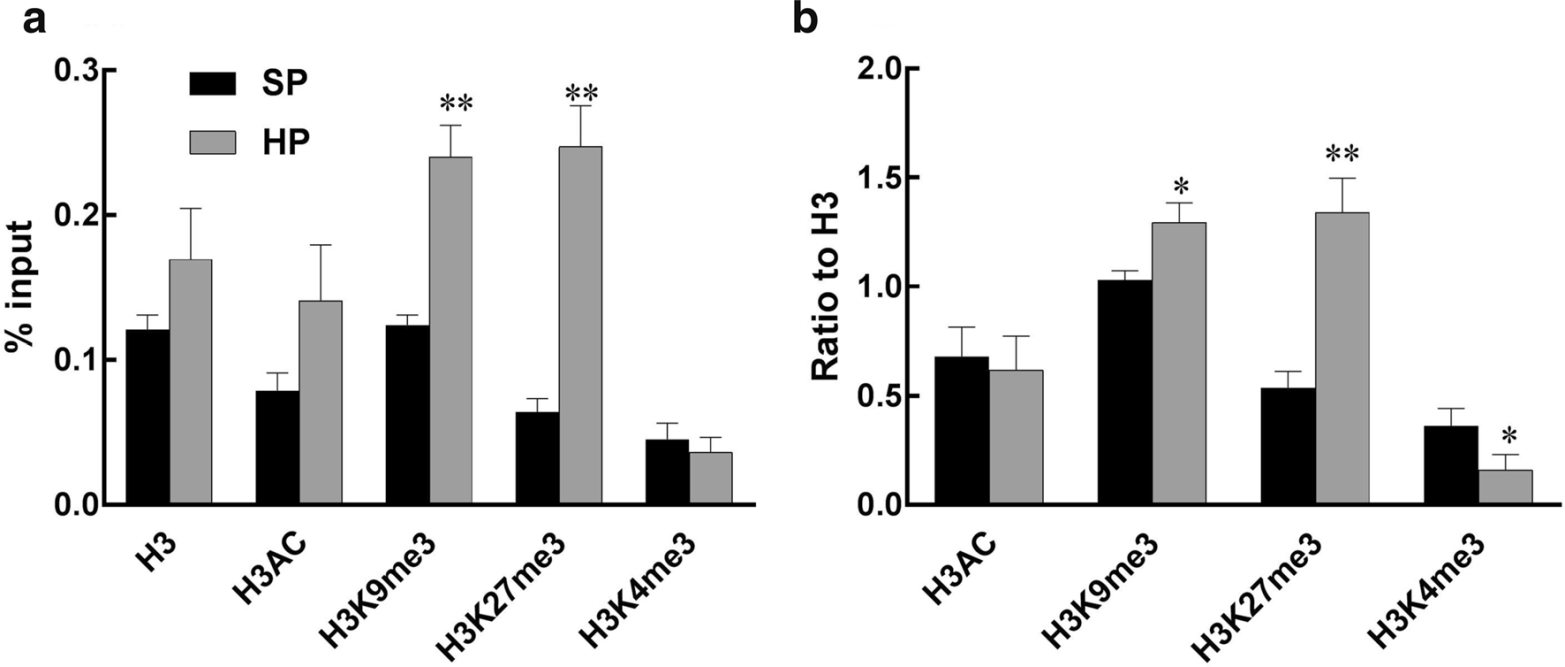
Chromatin immunoprecipitation (ChIP) analysis of histone marks binding to the IGFBP-3 gene promoter in the liver of weaning piglets. **a** normalized to input, **b** normalized to histone 3. Values are mean ± S.E.M., *n* = 14. * *p* < 0.05, ** *p* < 0.01

To determine the underlying mechanisms involved in the downregulated *IGFBP*-*3* gene expression in HP-exposed weaning piglets, MeDIP and ChIP-qPCR were performed to measure the methylation status of the promoter region of the *IGFBP*-*3* gene. In line with the change in the transcript level, DNA hypermethylation of the *IGFBP*-*3* gene was observed (Fig. 6). In addition, the repressive histone marks H3K27me3 and H3K9me3 were more highly enriched on the IGFBP-3 promoter in the livers of weaning piglets (Fig. 7a, b). Notably, the binding enrichment of the active histone mark H3K4me3 was also reduced when normalized to H3. Although histone acetylation is critical to regulate gene expression, H3AC was not significantly changed in response to HP exposure.

## Discussion

In this study, we presented evidence demonstrating that a high-protein diet during pregnancy led to higher body and liver weights in newborns, as well as in weaning piglets. Endocrine factors such as insulin, sex hormones and thyroid hormones play important roles in fetal development and growth. One study demonstrated that a maternal high-protein/low-carbohydrate diet increases the insulin resistance of animals to maintain glucose homeostasis without affecting the basal insulin level [35]. Accordingly, we reported that serum insulin levels were not changed in HP-exposed piglets. Sex hormones are considered an important factor and are thought to influence animal growth differently between males and females under maternal nutrition programming, but no sex disparity in body weight or hepatic growth axis was observed in our study regardless of treatment. Similarly, a human clinical trial revealed that postnatal diet affects sex steroid levels independent of infant size, differing from the prenatal nutrition intervention [36]. This finding suggests that neonatal growth after birth may not be mediated directly by sex hormones. Rather, we postulate that the changed IGF axis may be more sensitive to thyroid hormones. It has been reported that hypothyroidism is closely linked to body weight and the growth rate of offspring in early life [37–39]. Indeed, a recent study revealed that maternal high-protein diet consumption during pregnancy causes elevated body weight of offspring at birth without changing the IGF-I pathway [21]. Similarly, we demonstrated that T3 and T4 levels in the serum were higher in the HP-exposed piglets with higher body weight. We found that the increased body weight tended to diminish toward adulthood. This finding might be explained by the adaptation response, i.e., slower growth rate in the growing stage/fattening period of pigs [21]. Furthermore, we found that the IGF-1R protein content was increased, which was associated with elevated serum T3 levels at birth due to HP exposure. These results suggest that maternal high-protein intake promotes the activation of the IGF-I growth axis in the early life of offspring.

The IGF-IGFBP pathway is one of the most essential parts of growth axis regulation. Indeed, the regulation of IGF-dependent growth via maternal nutrition programming has been underlined, especially in IGUR models. In the current study, we demonstrated that liver IGF-1R protein content was increased at birth in response to high-protein exposure, indicating a stimulatory effect during the early life of offspring. This finding may explain the increased liver and body weights of neonatal and weaning piglets from the HP diet-fed sows compared to the weights of the control piglets. Interestingly, we found that at the weaning period (day 35), the IGFBP-3 levels in the serum and livers of the HP piglets were decreased. High-protein intake in pregnant women has been reported to be associated with reduced levels of IGF-II and IGFBP-3 in cord blood but to not affect IGF-I [40]. In contrast, it is demonstrated that nutrition restriction during pregnancy in ewes tended to decrease IGFBP-3 expression in the fetus while lowering plasma IGF levels [41, 42]. It is noteworthy that although IGFBP-3 was inclined to decrease, the IGFBP-2 level in fetal plasma was significantly increased due to maternal energy restriction [12]. Importantly, it has been described that during fetal life, the predominant IGFBPs are IGFBP-1 and IGFBP-2, whereas IGFBP-3 becomes the most abundant protein later in life, accounting for 80% of all IGF binding. Thus, this may explain the different results of our study on sows and the previous studies on dams and women compared to the nutrition restriction study in ewes [12].

On the other hand, it is widely accepted that poor fetal growth is associated with postnatal growth acceleration as a compensatory response and is linked to an increased risk of metabolic disorders in the long term [43, 44]. The early growth retardation and later life compensatory growth have also been observed after weaning and in adulthood [44, 45]. In this regard, the reduced IGFBP-3 expression reprogrammed by maternal high dietary protein may be an adaptation reaction to slow down growth from weaning to adult life. It is worth mentioning that the *IGFBP*-*3* gene, but not the protein content, was still downregulated until adulthood in our study. This implies that post-transcriptional regulation may be involved. One possibility is that microRNAs target mRNA degradation and/or translational repression. Numerous studies have reported that maternal dietary supplementation regulates gene translation by modifying microRNA function [31, 46]. However, whether the dissociation of the decreased *IGFBP3* mRNA and the unchanged IGFBP-3 protein expression in the liver of piglets is induced by a post-transcriptional mechanism is beyond the focus of the present study and may warrant future investigations.

The postnatal stage, especially the weaning period, is of critical importance for animal growth and for health in adult life [47, 48]. Based on our data and data from other studies, maternal nutrition programming modulates liver growth and function in offspring predominantly via epigenetic regulation [33, 49, 50]. Intriguingly, we demonstrated that the downregulated mRNA level of *IGFBP*-*3* in the liver was associated with protein content. Cancer research has shown evidence that the *IGFBP*-*3* gene is vulnerable to epigenetic regulation, including DNA methylation and histone modification [26]. However, the understanding of the maternal–offspring *IGFBP*-*3* gene expression regulation is limited. To support the notion that DNA methylation functions on the IGF axis in fetal growth [27], our data revealed that CpG methylation was enhanced on the promoter of *IGFBP*-*3* in HP piglets at weaning, thus blocking transcription to decrease *IGFBP*-*3* mRNA expression.

Having described the altered DNA methylation status, we further studied the mechanisms by conducting ChIP analysis of the *IGFBP*-*3* gene. We identified histone modification of the *IGFBP*-*3* gene promoter in the offspring exposed to maternal high-protein intake in pigs for the first time. Total histone H3 status represents a whole genomic histone methylation; however, it was not changed in the liver of weaning HP piglets. It is known that maternal nutrition supplementation modifies liver metabolic genes via the modification of histones, including H3K4me3, H3K27me3 and H3k9me1, in the offspring [29, 51]. For the specific gene *IGFBP*-*3*, enrichment changes in histone marks on the gene promoter region were in line with decreased *IGFBP*-*3* transcription. Higher enrichment levels of H3K9me3 and H3K27me3 were associated with DNA hypermethylation in the H piglets at weaning. This suggests that high protein content in the maternal diet plays a dominant role in gene methylation to epigenetically modulate the hepatic growth axis in offspring. Furthermore, histone acetylation is a pivotal factor that regulates fetal gene expression [52]. Although our data show that H3AC enrichment was not changed in this model, specific marks such as H3K27ac and H3K9ac should be measured in the future to confirm acetylation status in the offspring exposed to high-protein diet in pregnant dams.

In conclusion, our study reveals that a maternal high-protein diet given during gestation modulates the hepatic growth axis in weaning piglets. This may, at least partly, be attributed to the reprogramming of IGFBP-3 function by DNA methylation and histone modification. Our findings of epigenetic regulation of the IGFBP-3-mediated liver growth axis being involved in maternal nutrition programming provide an underlying mechanism of how dietary protein modulates fetal growth. Given that weaning is a critical period of life, a healthy growth pattern built from early life may reduce the risk of developing obesity in adulthood.

## Abbreviations

ChIP: Chromatin immunoprecipitation
E: Embryonic
GHR: Growth hormone receptor
HP: High protein
IGFBP-3: Insulin-like growth factor-binding protein 3
IUGR: Intrauterine growth retardation
LPD: Low-protein diet
MeDIP: Methylated DNA immunoprecipitation
MS: Meishan
PCR: Polymerase chain reaction
SDS: Sodium dodecyl sulfate
SP: Standard protein
